# Two successive outbreaks of acute gastroenteritis due to norovirus GII.6 in a holiday camp house

**DOI:** 10.1038/s41598-023-42622-z

**Published:** 2023-09-20

**Authors:** Miquel Alsedà, Pere Godoy, Pilar Bach, Núria Soldevila, Thais Cornejo, Laura Corominas, Maria Grau, Àngela Domínguez, The Working Group for the Study of Outbreaks of Acute Gastroenteritis in Catalonia, Miquel Alsedà, Josep Álvarez, Irene Barrabeig, Anna Isabel Belver, Neus Camps, Sofia Minguell, Monica Carol, Pere Godoy, Conchita Izquierdo, Mireia Jané, Ana Martínez, Ignacio Parrón, Cristina Pérez, Ariadna Rovira, Maria Sabaté, Maria Rosa Sala, Núria Torner, Rosa Maria Vileu, Anna de Andres, Javier de Benito, Esteve Camprubí, Montse Cunillé, M. Lluïsa Forns, A. Moreno-Martínez, Efrén Razquín, Cristina Rius, Sara Sabaté, Mercé de Simón, Virginia Rodríguez, Rosa Bartolomé, Thais Cornejo, Susana Guix, Lorena Coronas, Àngela Domínguez, Núria Soldevila

**Affiliations:** 1 Agència de Salut Pública de Catalunya, Lleida, Spain; 2 Institut de Recerca Biomédica de Lleida, IRB Lleida, Lleida, Spain; 3 CIBER Epidemiología Y Salut Pública (CIBERESP), Madrid, Spain; 4 Departament de Medicina, Universitat de Barcelona, Barcelona, Spain; 5 Laboratori de Microbiologia, Hospital Universitari Vall d’Hebrón, Barcelona, Spain; 6 Laboratori Salut Pública, Agència de Salut Pública de Catalunya, Girona, Spain; 7 Agència de Salut Pública de Barcelona, Barcelona, Spain; 8 Laboratori de Virus Entèrics, Universitat de Barcelona, Barcelona, Spain

**Keywords:** Infectious diseases, Epidemiology

## Abstract

When two outbreaks occur in the same institution within a short period of time, an important health and social concern is generated. Two gastroenteritis outbreaks occurring a week apart in the same facility were reported in Lleida, Spain, in 2018. The objective of this study was to describe the clinical, epidemiological and microbiological investigation carried out and to determine the risk factors. Demographic data, food consumption and symptoms were collected. Health inspections of the facility were carried out. Risk ratio and their 95% confidence intervals were estimated for the implication of each food consumed. The attack rate was 89.7% in the first outbreak and 69.6% in the second outbreak. The most frequent symptoms in the first and second outbreak were abdominal pain (88.5% and 100%, respectively), vomiting (80.8% and 87.5%, respectively) and nausea (69.2% and 81.3%, respectively). The first outbreak was associated with the consumption of a salad and the second with a cheese omelet. Norovirus GII.6 was detected by RT-PCR and sequenced in both groups of students and in the food handlers who prepared the meals. These results highlight the importance of exclusion from work of food handlers with gastroenteritis, the adequate availability of mechanisms for correct hand washing and the correct cleaning of surfaces.

## Introduction

Norovirus (NoV) is a very common cause of gastroenteritis worldwide^1,2^. NoV belongs to the *Caliciviridae* family, being genogroups (G) I, II and IV those that affect humans^3^. NoV infection results in both symptomatic cases and asymptomatic infections. The most characteristic clinical manifestations in symptomatic cases begin 24–48 h after exposure and consist mainly of diarrhoea, nausea and vomiting^4^. These symptoms usually last one to three days and most people recover without treatment^4^. In some cases, especially neonates, the elderly and immunosuppressed people, diarrhea can cause severe dehydration. Both symptomatic and asymptomatic individuals can act as transmitters, although asymptomatic individuals shed less virus. Viral shedding usually begins between 3 and 14 h before the onset of symptoms and generally reaches its peak five days after exposure^5,6^.

Infection occurs primarily via person-to-person transmission through the faecal-oral route, by aerosols produced by vomiting, or indirectly through contaminated food, water, or surfaces^7^. The cases of disease can appear grouped as outbreaks. In fact, NoV is considered the leading cause of acute gastroenteritis outbreaks^1,8^. A review of NoV gastroenteritis outbreaks pointed out that foodborne transmission and the venue where food was served were the most commonly associated factors^9^. In food, norovirus contamination can occur at the production point or during its preparation. Isolated outbreaks have been described in relation to different facilities^10–14^, but when several outbreaks occur in the same institution and within a short period, this generates significant health and social concern^15–17^. In these situations, it can be assumed either that the reservoir of the causal agent remains in the facility or that the causal agent has been reintroduced.

On 04/11/2018, the Epidemiological Surveillance Service of Lleida, Alt Pirineu and Aran (ESS) received the report of a possible outbreak of acute gastroenteritis in a group of students from a high school in Lleida who had attended a holiday camp house. A week later, on 04/18/2018, a possible second outbreak was reported at the same facility affecting another group from the same high school. The objective of this study is to describe the clinical, epidemiological and microbiological investigation carried out in both outbreaks of acute gastroenteritis and determine the risk factors associated to their occurrence.

## Materials and methods

### Study participants and data collection

#### Group 1

The first group was made up of 29 students and 3 teachers from a high school in the city of Lleida. The group arrived at the facility on 04/09/2018, on 04/10/2018 in the afternoon the first cases of gastroenteritis appeared and on 04/11/2018 the outbreak with 19 affected was reported. The clinical presentation of those affected was vomiting, abdominal pain, diarrhea and, in some cases, low-grade fever. Information on food consumption was as follows: at lunch on 04/09/2018, rice salad with mayonnaise sauce, breaded meat with French fries and tangerine; for dinner that same day, green beans, sausage and French fries, and yogurt. Breakfast on 04/10/2018 consisted of toast and ham. The same day's lunch consisted of a cheese sandwich, a chorizo sandwich and an apple taken out for picnic, where they drank water from a fountain in the Aigüestortes National Park.

#### Group 2

The second group was made up of 32 students and 3 teachers from the same high school who arrived at the facility on 04/16/2018, and first cases appearing the following day. This second outbreak was reported on 04/18/2018 with 12 affected presenting a similar clinical picture as in the previous group. Information on the consumed foods was as follows: at lunch on 04/16/2018, macaroni Bolognese, hake fillet with green salad and ice cream, and at dinner, green beans with potatoes, cheese omelet and yogurt. Breakfast on 04/17/2018 consisted of bread and butter, jam, sausage and pastries. That same day there was an excursion to the Aigüestortes National Park and picnic meal consisted of a ham sandwich, a sausage sandwich and an apple. They also drank water from a fountain in the Aigüestortes National Park.

According to data collected, the study hypothesis was the foodborne origin of two acute gastroenteritis outbreaks. From the ESS, a questionnaire was designed to collect information on demographic variables, food consumption, clinical symptoms presented and date of symptoms onset. The questionnaire was sent to the director of the high school, who distributed it among the group members. Subsequently, the questionnaires were collected and sent to the ESS for analysis. There were two foodhandlers in charge at the holiday camp house. Technicians from the ESS conducted telephone interviews with the food handlers to collect history or the presence of symptoms of acute gastroenteritis. The outbreak definition considered was the existence of two or more members of a group that presented gastrointestinal symptoms. The following case definition was established: student or teacher who presented vomiting and /or diarrhea or two or more of the following symptoms: abdominal pain, fever and nausea between 04/9/2018 and 04/11/2018 (group 1) or between 04/16/2018 and 04/18/2018 (group 2). A case was considered confirmed when the microbiological result rendered positive. The outbreak was reported to the Health Protection Service to proceed with the investigation of the food handling procedures followed and to carry out an inspection of the food safety conditions of the facility.

Stool samples were collected from those affected in each of the groups and from the food handlers. In food handlers, although they were the same, a first sample was obtained in the first outbreak and a second sample in the second outbreak. Fecal samples of outbreaks were analyzed in the laboratory of microbiology for the study of community outbreaks of food poisoning of the Vall d'Hebron University Hospital. The presence of the following enteropathogenic bacteria were investigated: *Salmonella, Shigella, Yersinia, Campylobacter* and *E. coli* O157 by means of conventional culture. Enteric viruses were detected using real-time PCR with the Allplex™ GI-Virus Assay (Seegene, Inc.), which simultaneously detects NoV GI, NoV GII, adenovirus, rotavirus, sapovirus and astrovirus. Samples positive for NoV GI and/or NoV GII were genotyped using GISKF-GISKR primers for GI and G2SKF-G2SKR for GII described by Kojima et al.^18^. Amplification was performed by RT-PCR using the One-Step RT-PCR kit (Qiagen, Hilden, Germany). Sequences were obtained using the ABI 3730 platform (Applied Biosystems, Foster City, California, USA) and were assembled with the SeqMan 4.05 program (Dnastar, Madison, WI, USA). Once the sequences were obtained, the genotype was obtained using the Norovirus Typing Tool Version 2.0 (https://www.rivm.nl/mpf/typingtool/norovirus/). Food samples were analyzed at the Girona Public Health laboratory. Detection of NoV GI and NoV GII was performed by RT-PCR using the One-Step RT-PCR kits (CeeramTools®, Biomérieux Spain). Thermocycler 7500 Fast Real-Time PCR System (Applied Biosystems) was used^19^.

### Statistical analysis

The survey coverage and attack rates, distribution of groups according to age and sex, epidemic curve, incubation period and frequency of symptoms were determined. The implication of each food consumed was performed by calculating the risk ratio (RR) and its 95% confidence interval (CI). When the contingency table presented a value of zero in a cell that prevented the calculation of the RR, 0.5 was added to each cell to calculate the RR^20^. Fisher's exact test was used to calculate the p-value.

### Ethics declarations and informed consent statement

The study was conducted according to the guidelines of the Declaration of Helsinki, regulations of the Public Health Agency of Catalonia and ethical protocols established. The study was approved by the University of Barcelona Bioethics Commission (ethics approval number IRB00003099) on April 12, 2016.

The authors declare that the Bioethics Committee of University of Barcelona approved the waiver for informed consent. All data used in the analysis were collected during routine public health surveillance activities as part of the legislated mandate of the Health Department of Catalonia, which is officially authorized to receive, treat and temporarily store personal data in the case of infectious disease. All data were fully anonymized. All study activities formed part of the public health surveillance tasks. The law regulates these activities and informed consent should not be necessary.

## Results

**Figure 1:**
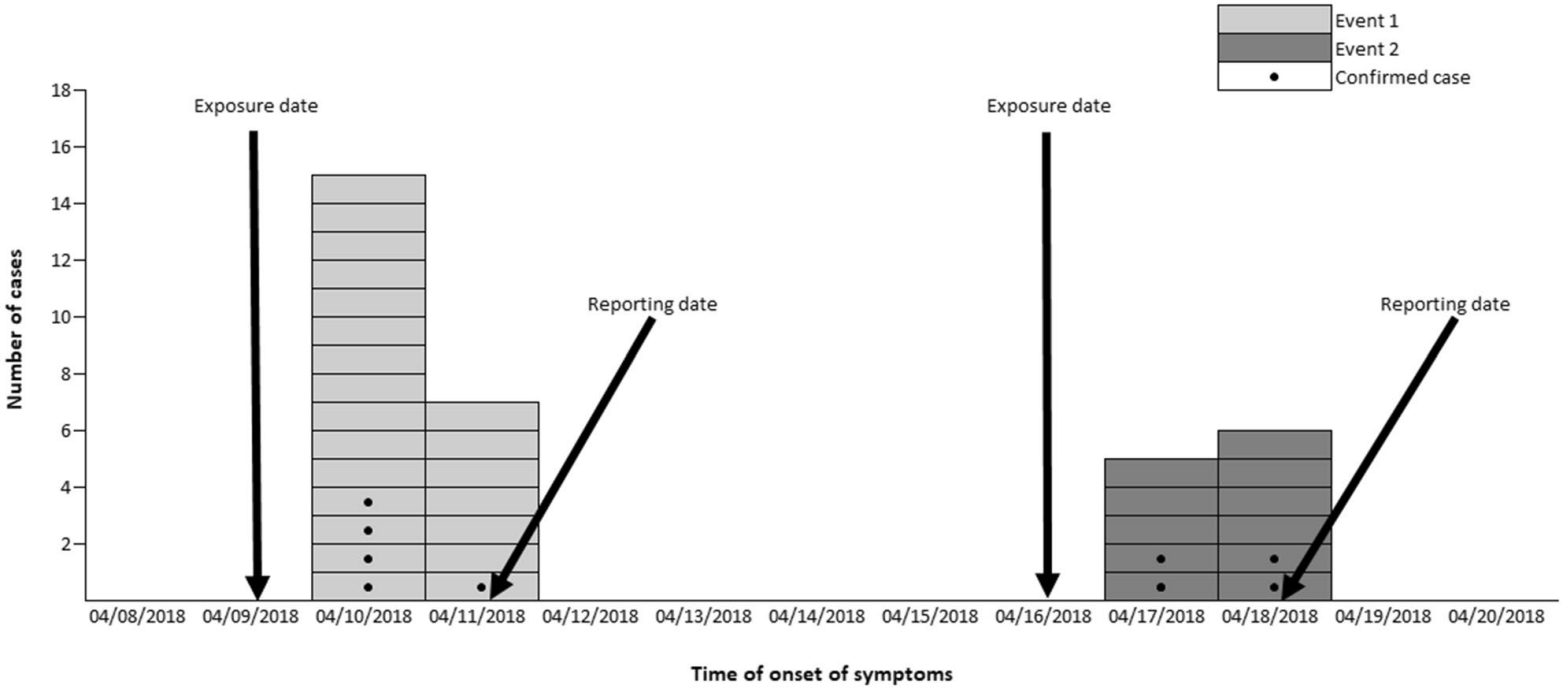
Epidemic curve for the occurrence of the first and second event.

In the first outbreak, 29 surveys of the 32 members of the group were analyzed (coverage rate of 90.6%). Among those studied, attack rate was 89.7% (26/29). The age of the students was 12–13 years old (21 and 5, respectively); teachers (3) were between 43 and 53 years old. In relation to gender, 51.7% (15/29) were men. The cases started symptoms after 5:00 p.m. on 04/10/2018, 15 cases appeared that day and 7 the next day; there were 4 cases in which the onset date was not obtained (Fig. 1). The symptoms presented were abdominal pain in 88.5% (23/26), vomiting in 80.8% (21/26), nausea in 69.2% (18/26), and diarrhea and fever in 50% (13/26). Food stuff that showed a higher risk ratio was the green salad (RR = 4.00; 95% CI 0.71–21.84; *p*-value < 0.01) (Table 1). Fixing the point of exposure at consumption of the salad, the incubation period of 22 cases was calculated. The median of incubation period was 31 h (range 27h-46h).

**Table 1:** Analysis of food and beverages involved as a vehicle of transmission in the first outbreak.

Foods and beverages	Consumers				Non consumers				RR (95% CI)	*p*-value
	Cases	Non cases	Total	Attack rate (%)	Cases	Non cases	Total	Attack rate (%)		
Natural Park fountain water *	11	1	12	91.7	13	2	15	86.7	1.06 (0.81–1.37)	1.00
Chorizo sandwich	13	0	13	100	13	3	16	81.3	1.23 (0.97–1.56)	0.31
Cheese sandwich	13	3	16	81.3	13	0	13	100	0.81 (0.64–1.03)	0.31
Vegetable salad with mayonnaise**	24	0	24	100	1	3	4	25	4.00 (0.71–21.8)	< 0.01
Ham sandwich	12	0	12	100	14	3	17	82.4	1.21 (0.97–1.51)	0.37
Green beans	19	3	22	86.4	7	0	7	100	0.86 (0.73–1.02)	0.84
Tangerine	15	2	17	88.2	11	1	12	91.7	0.96 (0.75–1.23)	1.00
Apple	7	1	8	87.5	19	2	21	90.5	0.96 (0.72–1.30)	1.00
Sausage and potatoes	23	3	26	88.5	3	0	3	100	0.88 (0.77–1.02)	1.00
Breaded ham and cheese	24	3	27	88.9	2	0	2	100	0.89 (0.78–1.02)	1.00
Toast	16	1	17	94.1	10	2	12	83.3	1.13 (0.85–1.49)	0.73
Yogurt**	23	3	26	88.5	2	0	2	100	0.88 (0.77–1.02)	1.00

RR (95% CI): Risk ratio and 95% Confidence interval.

*Information on its consumption was obtained from 27 respondents.

**Information on its consumption was obtained from 28 respondents.

Fecal samples were collected from 10 cases for whom results were negative in the investigation of enteropathogenic bacteria, yet virological analysis detected NoV GII.6 in 5 of them. An analysis of a sample of the testimonial menu of the salad was carried out with negative result to NoV GI and GII**.** No investigation was conducted on the park water because in the first group there was no association (RR = 1.1; 95% CI 0.8–1.3; *p*-value = 0.95).

The two food handlers had suffered from gastroenteritis with symptom onset on the previous days (04/08/2018 and 04/10/2018, respectively), although they had already recovered when the survey was conducted. Fecal samples were collected from both food handlers with negative results in the enteropathogenic bacteria investigation and in the virological study. The food safety inspection of the facility recommended equipping the sinks at the toilets and handwashing points in the kitchen, and to provide soap dispensers and paper towels. Extreme measures for hygiene on kitchen surfaces and utensils were urged and food handlers were reminded that in case of gastroenteritis they should be absent from their workplace for up to 48 h after symptoms resolve.

In the second outbreak, the coverage rate was 65.7% (23/35). In this group, no surveys were obtained from teachers. The attack rate was 69.6% (16/23). The age of the respondents was 12–13 years (10 and 6, respectively); 47.8% (11/23) of the respondents were men. The first cases started symptoms on 04/17/2018 (5 students) and 6 cases did so the next day; in 5 cases, this information was not obtained (Fig. 1). Symptoms were abdominal pain in 100% (16/16), vomiting in 87.5% (14/16), nausea in 81.3% (13/16), and diarrhea and fever in 68.8% (11/16). The food that showed a higher risk ratio was the cheese omelet with a RR = 2.87; 95% CI 0.26–32.08 (*p*-value = 0.19) (Table 2). Placing the source of exposure at the consumption of omelet point, the incubation period of 15 cases was calculated. The median of incubation period was 28 h (range 16h-49h). Stool samples were collected from 9 cases with negative results for enteropathogenic bacteria, and positive NoV GII.6 detected in 44.4% (4/9) of them. An analysis of the cheese omelet sample was not possible because there was no testimonial menu.

**Table 2:** Analysis of food and beverages involved as a vehicle of transmission in the second outbreak.

Foods and beverages	Consumers				Non consumers				RR (95%CI)	*p*-value
	Cases	Non cases	Total	Attack rate (%)	Cases	Non cases	Total	Attack rate (%)		
Natural Park fountain water	2	0	2	100	14	7	21	66.7	1.50 (1.11–2.03)	0.95
Chorizo sandwich	11	3	14	78.6	5	4	9	55.6	1.41 (0.74–2.70)	0.48
Ham sandwich	12	3	15	80	4	4	8	50	1.60 (0.77–3.35)	0.31
Peperoni sandwich	12	3	15	80	4	4	8	50	1.60 (0.77–3.35)	0.31
Pastry*	8	3	11	72.7	6	4	10	60	1.21 (0.65–2.26)	0.88
Ice cream	14	7	21	66.7	2	0	2	100	0.67 (0.49–0.90)	0.95
Green beans and potatoes	15	7	22	68.2	1	0	1	100	0.68 (0.51–0.91)	1.00
Macarroni	15	7	22	68.2	1	0	1	100	0.68 (0.51–0.91)	1.00
Apple	8	5	13	61.5	8	2	10	80	0.77 (0.45–1.31)	0.63
Hake	14	6	20	70	2	1	3	66.7	1.05 (0.45–2.46)	1.00
Bread and butter with marmalade**	10	3	13	76.9	5	4	9	55.6	1.39 (0.72–2.67)	0.55
Cheese omelet	16	6	22	72.7	0	1	1	0	2.87 (0.26–32.08)	0.19
Yogurt*	12	4	16	75	3	2	5	60	1.25 (0.58–2.70)	0.90

RR (95%CI): Risk ratio and 95% Confidence interval.

*Information on its consumption was obtained from 21 respondents.

**Information on its consumption was obtained from 22 respondents.

The two food handlers were surveyed again and both stated that they had not suffered a new episode of acute gastroenteritis. Fecal samples collected from them were negative to bacteriological investigation, but positive for the virological study, detecting NoV GII.6 in both. In the health inspection after the second outbreak, as in the previous inspection, the sinks in the toilets and handwashing points in the kitchen had not been equipped with soap dispensers and paper towels. A lack of adherence to the indications for disinfectant to be used to clean surfaces was also observed, and a specialized company was requested to disinfect the kitchen and toilets.

## Discussion

The investigation of two outbreaks of gastroenteritis that affected the same facility occurring within a one week time span is presented. The attack rate of the first outbreak was 89.7% (26/29) and that of the second 69.6% (16/23). In both outbreaks, the predominant symptoms were abdominal pain and vomiting. In the first outbreak, the consumption of green salad was implicated and in the second, it was the consumption of cheese omelet. The stool samples of those affected were positive to NoV GII.6 in both the first and second outbreaks. The analyses of stool samples performed on the facility’s food handlers, in the first outbreak were negative for enteropathogenic bacteria and viruses and in the second outbreak both food handlers were positive for NoV GII.6.

The impact of both outbreaks on school groups was high, presenting an attack rate close to 90% in the first group and 70% in the second, with high survey coverage rates (90.6% in the first outbreak and 65.7% in the second). In a review of 902 outbreaks of gastroenteritis due to NoV^9^, it was concluded that outbreaks associated with transmission through food consumption were those with the highest attack rates, followed by those with transmission through water and then those with human transmission person to person. In our same geographical area, a waterborne NoV outbreak affecting 95 schoolchildren was caused by the consumption of water coming from the center's own tank that was fed by the municipal network, presented an attack rate of 45%^21^. In a recent publication that exposes the outbreaks caused by norovirus in nursing homes in Catalonia in the period 2017–2018^22^, 27 outbreaks of person-to-person transmission were presented and the global attack rate was 25% among residents and 11% among the staff. The study by Chan et al.^23^ also suggests that people with a G II NoV infection could shed a higher concentration of the virus than those infected with G I. In our study, both the most likely transmission mechanism and the causative agent could explain the high attack rates detected.

The symptoms of those affected in both outbreaks were similar, highlighting a clear predominance of abdominal pain and vomiting; the percentage of cases with fever was 50% in the first outbreak and 68.8% in the second. This clinical presentation would be related to the age of those affected. Thus, in a study on the clinical characteristics of those affected in outbreaks of gastroenteritis by NoV^24^ in which 1,544 cases were analyzed, it was highlighted that children and adolescents present vomiting more frequently, unlike adults who suffer from diarrhea as the most frequent symptom. In an outbreak caused by foodborne norovirus GII.6^25^ that affected schoolchildren aged 9 and 10 years, the frequency of vomiting was 100%, diarrhea 56.3%, and fever 15.6%. Studying a cohort of 900 children, Bhavanam et al.^26^ found that, although the frequency of vomiting and diarrhea was similar for the GI and GII genogroups, those affected by GII more frequently presented febrile symptoms and a longer clinical course.

The epidemic curves of both outbreaks are typical of a point exposure in a common source outbreak. The large clustering of cases and their rapid increase and decrease suggest a point source exposure through a common vehicle. In the epidemiological analysis to identify the foods and beverages associated with the outbreak, there was one food detected in each one of the outbreaks as the most likely to be involved (the salad in the first outbreak and cheese omelet in the second). The RR for the food involved in the second outbreak was not statistically significant. However, in the second outbreak, the cheese omelet showed one of the highest attack rates among consumers and was the only food that was not associated with cases among non-consumers. The analysis of the testimonial menu sample from the salad was carried out, but the result was negative to NoV GI and GII. Yet, this negative result does not rule out the involvement of the salad since the sensitivity of NoV detection in food is low, either due to a low concentration of the virus in the food and/or the existence of inhibitory factors^27^. It is also possible that the virus was not homogeneously distributed in the food.

Five samples positive to NoV GII.6 were detected among cases in the first outbreak and four in the second. Although the food handlers' samples were negative in the first outbreak, they were positive for NoV GII.6 in the second. In all samples, the bacteriological investigation for enteropathogenic bacteria was carried out, with negative results in all of them. Based on all the results obtained, it can be ascertained that NoV GII.6 was the etiological agent that caused both outbreaks. In a review of 493 foodborne outbreaks detected in the United States^28^, 93 outbreaks (19%) were caused by NoV GI and 400 (81%) were caused by NoV GII, with the most frequent genotypes being GII.4 258 (52%), GII.6 45 (9%) and GI.3 38 (8%). In a recent study of acute NoV gastroenteritis in children worldwide^29^, GII.4 Sydney was the most frequent genotype detected, 52% (687/1325), followed by GII.3, 14% (190/1325); GII.2, 11% (149/1325) and GII.6, 5% (64/1325).

The involvement of food handlers in the first outbreak seems obvious. Although their stool samples were negative in the virological study, the epidemiological survey found that the first handler stated having had acute gastroenteritis symptoms the day before the arrival of the first school group and the second handler the day after their arrival. However, in the second outbreak, they did not report having had a new episode of acute gastroenteritis neither during the period between the two outbreaks nor after, yet the samples analyzed after the notification of the second outbreak were positive for NoV GII.6 in the samples collected from both food handlers. These results could be compatible with those obtained by some authors^30,31^ who state that those affected by gastroenteritis can excrete NoV in their feces for long periods of time, but with some degree of irregularity. In addition, the results of the minimum incubation periods in the first outbreak (27 h) and in the second (16 h) suggest that in both outbreaks NoV was acquired in the facility. Former results as well as the features of both epidemic curves could rule out an external introduction of the virus transmitted by the incoming school groups.

The sanitary inspection of the kitchen and toilets of the center showed the existence of contributing factors for the occurrence of both outbreaks. The lack of soap dispensers and paper towels at the toilet sinks and handwashing points in the kitchen would be some of them. In addition, in the inspection carried out because of the second outbreak, these deficiencies had not yet been corrected. A lack of adherence to the guidelines for the proper use of disinfectant in cleaning surfaces given by the inspection team was observed. A limitation of the present study is that surface samples were not studied in this outbreak, so the possible involvement of contaminated surfaces cannot be ruled out. Another limitation is that no genomic characterization assessment was carried out on the positive NoV samples.

## Conclusions

In conclusion, the results of the investigation of these outbreaks show that, in order to avoid the occurrence of successive outbreaks in the same facility, the exclusion from work of food handlers who present symptoms of gastroenteritis, up to 48 h after resolution of symptoms, is of great importance. It is also necessary to make adequate devices for proper hand washing (soap dispensers and hand dryers) available everywhere. Finally, another key factor would be the correct cleaning of surfaces with adequate adherence to disinfectant use guidelines since NoV has high environmental survival rate and can remain infective for more than two weeks on surfaces^32^.

## Data Availability

The datasets generated during and/or analyzed during the current study are available from the corresponding author on reasonable request.
